# Soy, milk and wheat allergy

**DOI:** 10.1186/2045-7022-1-S1-S39

**Published:** 2011-08-12

**Authors:** Itu Komei

**Affiliations:** 1 Aichi Children's Health and Medical Center, Department of Allergy, Obu, Japan

## 

Food allergies affect 5-10% of infants, 2-5% of toddlers, and 1.3-2.6% of school children in Japan. Egg, milk and wheat are the major three food allergens. Soybean is one of the eight foods causing immediate allergic reactions, sometimes anaphylaxis. Fermented soybeans, miso and shoyu, are used in most of the traditional Japanese foods, and exposure to soybeans in Japanese children begins as one of the first solid foods, typically in the form of tofu. Sensitization to soy, milk and wheat occurs in many atopic babies. Some are the primary sensitizations to the foods through oral or cutaneous route, but others may be the secondary sensitization through cross-reactive foods or pollens.

Component-specific IgE tests provide us more sensitive and specific diagnostic tools. We have found that casein, one of the most classically known allergen components in milk, can be a more specific marker in the diagnosis of milk allergy, especially in the older children. Recombinant ω-5 gliadin ImmunoCAP® provides almost 100 % positive predictive value of immediate type wheat allergy in children at 17.5 kUA/L, as well as wheat-dependent exercise-induced anaphylaxis in adults. Gly m 5 and Gly m 6 from soybean can be a marker of severe primary soy allergy in Japanese children. On the other hands, patients with secondary soy allergy in adults, who have allergic reactions exclusively to soy milk, but not to tofu, are predominantly sensitized to Gly m 4 (PR-10), possibly due to the cross-reactive pollen allergens.

In conclusion, allergen component testing is an excellent clinical tool from a diagnostic point of view but also provide us with new insights into food allergy.

